# Deciphering the underlying mechanism of Xianlinggubao capsule against osteoporosis by network pharmacology

**DOI:** 10.1186/s12906-020-03007-1

**Published:** 2020-07-03

**Authors:** Hangsheng Bao, Huizhi Guo, Zongquan Feng, Xin Li

**Affiliations:** 1 Foshan Hospital of Traditional Chinese Medicine, Foshan, 528000 China; 2 First Clinical Medical College, Guangzhou University of Chinese Medicine, Guangzhou, 510405 China; 3 Department of Nephrology, The First Affiliated Hospital of Guangzhou University of Chinese Medicine, Guangzhou, 510405 China

**Keywords:** Xianlinggubao capsule, Network pharmacology, Osteoporosis

## Abstract

**Background:**

Xianlinggubao formula (XLGB), a Chinese State Food and Drug Administration-permitted traditional Chinese herbal medicine, has been extensively used to treat osteoporosis. Although XLGB was shown to improve bone mass in ovariectomized rats and clinically alleviate osteoporosis symptoms, its pharmacological mechanisms remain unclear.

**Methods:**

In this study, we used a network pharmacological approach to explore the potential mechanism of XLGB in treating osteoporosis. We obtained XLGB compounds from the TCMSP and TCMID databases and identified potential targets of these compounds through target fishing based on the TCMSP and Swiss Target Prediction databases. Next, we identified the osteoporosis targets by using the CTD, TTD, GeneCards, OMIM and PharmGKB databases. Then, the overlapping genes between the XLGB potential targets and the osteoporosis targets were used to establish a protein-protein interaction (PPI) network and to analyze their interactions and identify the major hub genes in this network. Subsequently, the Metascape database was utilized to conduct the enrichment of Gene Ontology biological processes and Kyoto Encyclopedia of Genes and Genomes (KEGG) pathways.

**Results:**

There were 104 active compounds and 295 related targets identified overall. After the Metascape enrichment analysis, we identified the top 25 cellular biological processes and top 15 pathways based on the logP value and found that the XLGB-mediated anti-osteoporosis effect was mainly associated with reactive oxygen species, organonitrogen compound response and cell migration. Furthermore, 36 hub genes of XLGB, such as EGF, EGFR, MTOR, MAPK14 and NFKB1, were considered potential therapeutic targets, suggesting the underlying mechanisms of XLGB acting on osteoporosis.

**Conclusion:**

We investigated the possible therapeutic mechanisms of XLGB from a systemic perspective. These key targets and pathways provide promising directions for future research to reveal the exact regulatory mechanisms of XLGB.

## Background

Osteoporosis (OP) is a systemic bone disorder characterized by low bone mass and an accompanying increased incidences of fractures. And OP is usually considered to be the result of an imbalance between osteoclasts promoting bone absorption and osteoblasts inducing bone remodeling [1]. A recent study noted that over 10 million people suffered from osteoporosis in the United States. Moreover, its treatment burden, $22 billion in 2008, is expected to rise due to the consistently increasing aging population. Related osteoporosis treatments should be used to alleviate or reduce symptoms such as fractures, which could result in substantial cost savings [2]. For inhibition of bone loss and maintenance of bone mass, antiresorptive agents, anabolic agents and bone mineral drugs have been widely used in clinical treatment [3]. However, notably, almost all these anti-osteoporosis methods have limitations and side effects, such as increasing the risk of breast cancer, jaw necrosis or atypical femur fracture [4, 5]. Therefore, the development of alternative and safe intervention strategies for osteoporosis is needed.

Traditional Chinese medicine (TCM) has attracted worldwide attention and served as a main alternative treatment in East Asia, North America, and Europe due to its satisfactory curative effect, relatively low toxicity and low cost [6–8]. TCM has been used in China for thousands of years and has been shown to have good therapeutic effects and few side effects through multiple herb combinations for the prevention and treatments of various diseases [9]. Furthermore, an increasing number of TCMs have been found to be effective in the treatment of osteoporosis [10, 11].

As an important type of TCM, Xianlinggubao formula (XLGB) has been proven to effectively improve bone mass in ovariectomized rats [12] and clinically alleviate osteoporosis symptoms [13]. A multicenter and double blind clinical trial confirmed its beneficial effect on postmenopausal women with osteoporosis as well [14]. XLGB, similar to other TCM formulas, consists of six herbs with percentages in weight: *Epimedium sagittatum* Maxim (70%), *Dipsacus inermis* Wall. (10%), *Salvia miltiorrhiza* Bunge (5%), *Anemarrhena asphodeloides* Bunge (5%), *Psoralea corylifolia* L. (5%), and *Rehmannia glutinosa* Steud. (5%) [12]. Due to its therapeutic efficacy and safety, the Chinese State Food and Drug Administration (CFDA) has officially permitted XLGB capsules to be sold as an over-the-counter (OTC) drug for osteoporosis treatment [14]. Although XLGB performed well in clinical practice, its exact therapeutic mechanism in osteoporosis is still unclear. Similar to all TCM decoctions, XLGB exerts its therapeutic efficacy by regulating multiple molecules in the human body. Therefore, it is still a major challenge to identify its mechanism through scientific trials that are used in Western medicine. Fortunately, the development of systems pharmacology offers researchers an alternative opportunity and option to investigate the pharmacological mechanisms of TCM [15]. Recently, Wang et al. utilized a network pharmacology method to clarify the synergistic mechanism of Er-Xian decoction in treating osteoporosis [16]. Similarly, Zhang et al. employed this systems pharmacology method to dissect the mechanisms underlying the therapeutic effect of *Dipsacus inermis* Wall. on osteoporosis [17].

**Fig. 1:**
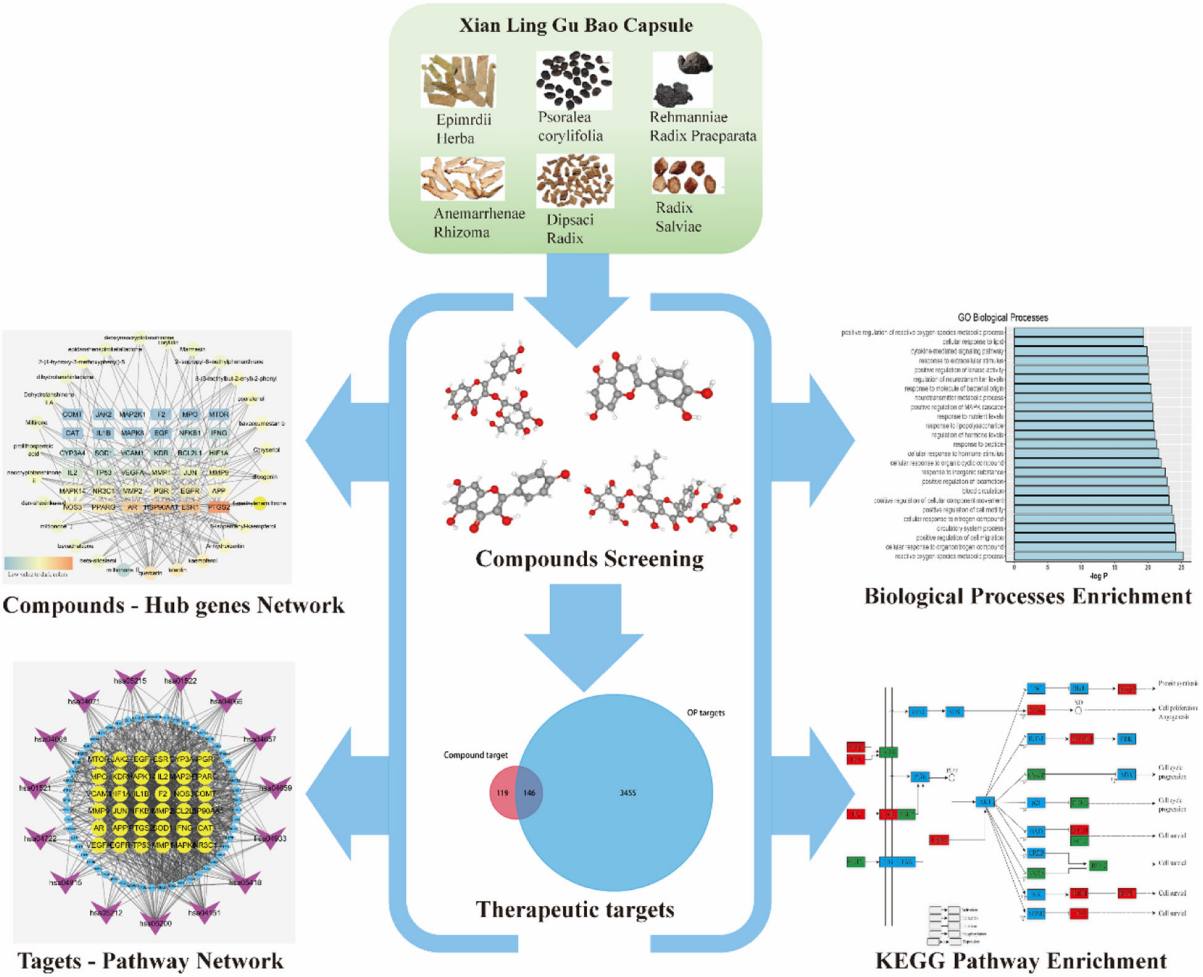
The schematic map of network pharmacology for investigating potential mechanisms of XLGB capsule in the OP treatment

In this network pharmacology study, we aimed to comprehensively dissect the mechanisms of XLGB in treating osteoporosis. We identified compounds related to the XLGB capsules based on multiple databases and obtained the compounds’ potential targets via target fishing. Then, we matched them with osteoporosis-related targets collected through a multisource database. Next, using overlapping targets from the previous process, we built a protein-protein network to analyze their internal interactions and then identified the hub genes. Furthermore, we used the Metascape database to conduct the biological process and Kyoto Encyclopedia of Genes and Genomes (KEGG) pathway enrichment analyses. The protocol of our experimental procedures is shown in Fig. 1.

## Methods

### Identification of chemical ingredients

To establish a compound ingredient database, we utilized the Traditional Chinese Medicine System Pharmacology Database (TCMSP™, http://lsp.nwu.edu.cn/tcmsp.php) [18], which is a frequently used platform for systems pharmacology. The Traditional Chinese Medicines Integrated Database (TCMID, http://119.3.41.228:8000/) [19] is one of the largest comprehensive TCM platforms. In total, one thousand and seventy-four chemical ingredients were identified in this part.

### Screening of active ingredients

#### OB evaluation

Oral bioavailability (OB) refers to the percent of an orally administered drug that reaches systemic circulation and is one of the most important pharmacokinetic indexes for drug screening. The TCMSP platform has adopted the OBioavail1.1 system, which integrates P450, 3A4 and P-glycoprotein information to obtain the OB value [20]. To determine the active ingredients used for further steps, we set the OB threshold at 30%.

#### DL prediction

Drug-likeness (DL) is a qualitative index that represents the “drug-like” degree of the target compound and is used to remove chemically unsuitable compounds. TCMSP used the Tanimoto similarity method to calculate the DL index by comparing the target compound to all 6511 molecules in the DrugBank database [21]. In this process, the compounds that did not meet the condition that DL ≥ 0.18 were removed. For herbs that were not included in TCMSP, we used the Swiss ADME database (http://www.swissadme.ch/) [22] to conduct the screening process. This platform allowed us to compute the pharmacokinetic properties and drug-like nature of the target compounds. This database has five different rule-based filters to define how drug-like the compound is. All these filters, including Lipinski, Ghose, Veber, Egan and Muegge, are from major pharmaceutical companies aiming to improve the quality of their drug development [22]. Thus, the compounds that met at least four rules were considered active ingredients and used for the next steps.

#### Caco-2 permeability

The permeability of the target drug is another critical part of drug development. The participation of villi and microvilli strongly increases the small intestinal absorption of orally administered drugs. Thus, the TCMSP platform used the Caco-2 permeability model based on 100 drug molecules with satisfactory statistical results (R2 > 0.8) to find compounds with good permeability. Therefore, we set the Caco-2 threshold at 0, and the compounds that met these criteria were used for further experiments.

### Target prediction of active ingredients

The compound ingredients have effects on the targets to exert their biological function. Thus, we used the TCMSP platform to predict the targets of active ingredients. For herbs that were not included in TCMSP, we used the Swiss Target Prediction database instead (http://www.swisstargetprediction.ch/index.php). After obtaining canonical descriptors of ingredients, we assessed their potential targets via this webserver based on chemical similarity.

### Disease target identification by multiple databases

We used multiple databases to collect OP-related targets, and the term ‘Osteoporosis’ was searched as the key word. The databases in this step included the Comparative Toxicogenomics Database (CTD, http://ctdbase.org/) [23], GeneCards (https://www.genecards.org/) [24], the Therapeutic Target Database (TTD, https://db.idrblab.org/ttd/) [25], OMIM (https://www.omim.org/) [26] and PharmGKB (https://www.pharmgkb.org/) [27]. In total, three thousand six hundred and one OP target were found through these databases.

Then, we constructed a Venn diagram to determine the overlapping targets between the OP and active ingredient targets because these overlapping targets play a critical role when XLGB treats OP. These targets were analyzed by String (https://string-db.org/) [28], and the protein-protein interaction (PPI) data were exported.

### Network construction

#### Construction method

Three main networks were built in this process, including 1. The compound-target network, 2. The XLGB target-OP target interaction network and 3. The target-pathway network. Target information was obtained from the KEGG pathway enrichment results. Cytoscape 3.6.2 (http://www.cytoscape.org/) was used in all network visualizations and is one of most powerful open-source software programs for constructing different networks visually [29]. However, there are several limitations in this network pharmacology study, as we did not determine whether the compounds activate or inhibit the targets, how they mediate the effect on the targets, binding or catalysis, and so on.

#### Topological feature definition

We used three parameters to describe and quantify the importance of nodes in these networks because the nodes that bridge many edges with neighborhoods are more likely to exert crucial mediating functions. 1. ‘Degree’ indicates the number of edges with other nodes. Examining the node’s degrees is the most straightforward method of quantifying the node centrality [30]. 2. “Betweenness centrality” is used to measure code centrality based on the shortest paths. A high value node would play a more important role in the network because more regulation information will pass through it [31]. 3. “Closeness centrality” represents the mean distance between the node and all other nodes in the network and is the reciprocal of the sum of the length of the shortest paths between itself and all other nodes in the network. Therefore, the central node would be more likely to be close to all other nodes [32].

### Biological process and pathway enrichment analysis

We used the Metascape database (http://metascape.org/gp/index.html) [33] to conduct the Gene Ontology (GO) biological process enrichment analysis and conducted it based on Kyoto Encyclopedia of Genes and Genomes [34] (KEGG, http://www.kegg.jp/) data obtained from Metascape.

## Results

### Active compounds of the XLGB capsules

**Fig. 2:**
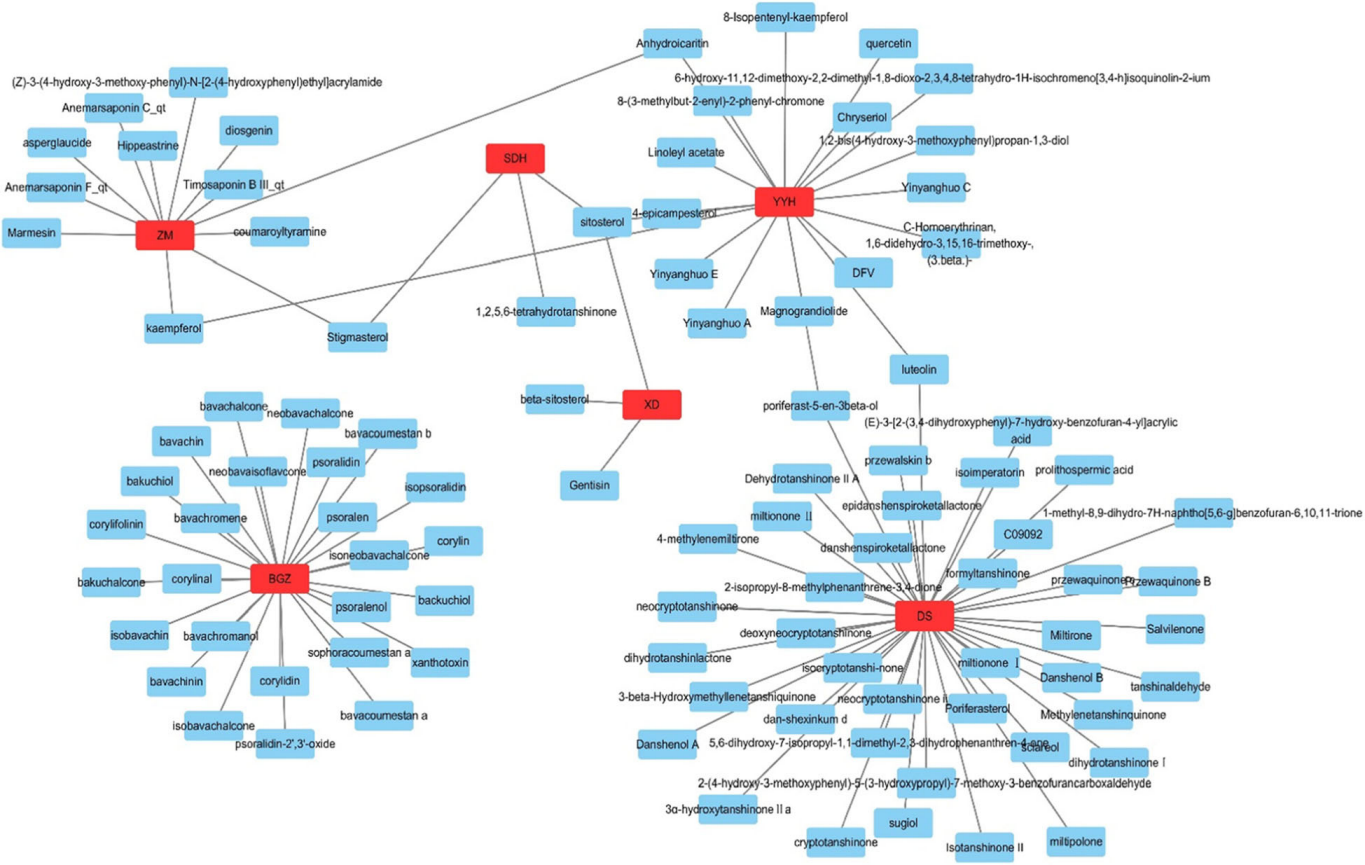
The network of Herbs-Compounds connection. The red nodes represent ingredients in XLGB capsule while the blue ones represent active compounds

From the TCMSP and TCMID databases, we identified 553 related compounds in the formula, among which there were 130 ingredients (23.5%) in Yin Yang Huo (*Epimedium sagittatum* Maxim, YYH), 202 (36.5%) in Dan Shen (*Salvia miltiorrhiza* Bunge*,* DS), 76 (13.7%) in Di Huang (*Rehmannia glutinosa* Steud, DH), 31 (5.6%) in (*Dipsacus inermis* Wall.*,* XD), 81 (14.6%) in Zhi Mu (*Anemarrhena asphodeloides* Bunge, ZM), and 33 (6.0%) in Bu Gu Zhi (*Psoralea corylifolia* L.*,* BGZ). After screening by the ADME thresholds (OB ≥ 30%, DL ≥ 0.18 and Caco-2 > 0) and five different rule-based filters of the Swiss ADME database, we obtained 104 active compounds. Then, the herb-compound network was constructed as shown in Fig. 2. Subsequently, we analyzed and reordered these compounds in descending order of edge betweenness, and the top three compounds were sitosterol (OB = 36.94, DL = 0.75, Caco-2 = 1.32), luteolin (OB = 36.16, DL = 0.25, Caco-2 = 0.19) and stigmasterol (OB = 43.83, DL = 0.76, Caco-2 = 1.44).

### Target prediction and analysis

**Fig. 3:**
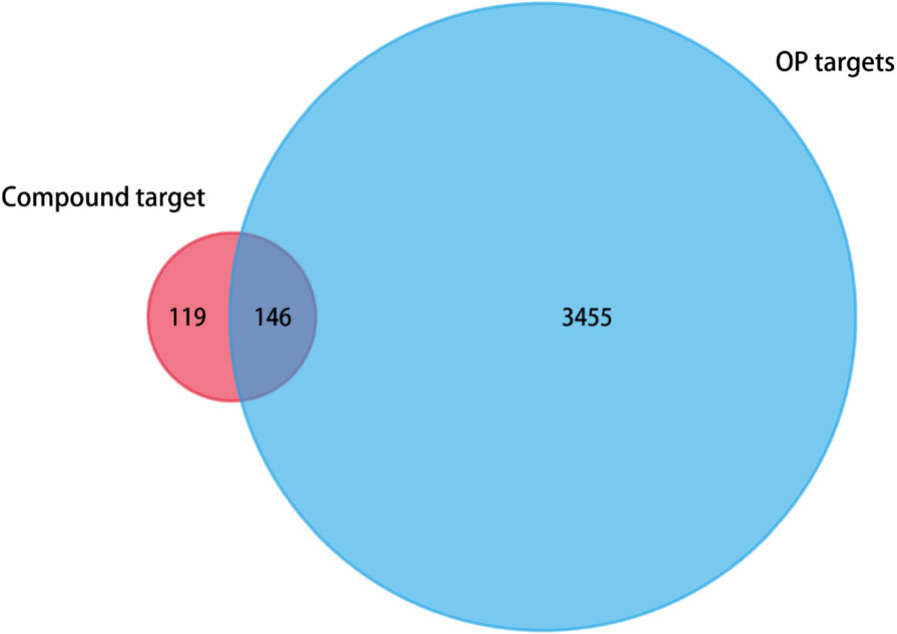
The venn diagram for compound and OP targets. The overlap targets mean the potential therapeutic gene for XLGB when treating OP

Next, we conducted target fishing for these 104 active ingredients using the TCMSP and Swiss Target Prediction databases based on chemical similarity. We obtained 295 related targets, among which BGZ had 191 targets, DS had 67 targets, DH had 27 targets, XD had 38 targets, YYH had 110 targets and ZM had 79 targets. We integrated the OP genes obtained from multisource databases, including CTD, GeneCards, TTD, OMIM and PharmGKB, and a total of 3601 related genes were identified After the construction of the Venn diagram, one hundred forty-six overlapping targets between the related targets of XLGB and OP were selected as the key targets through which XLGB exerts an anti-OP effect (Fig. 3).

**Fig. 4:**
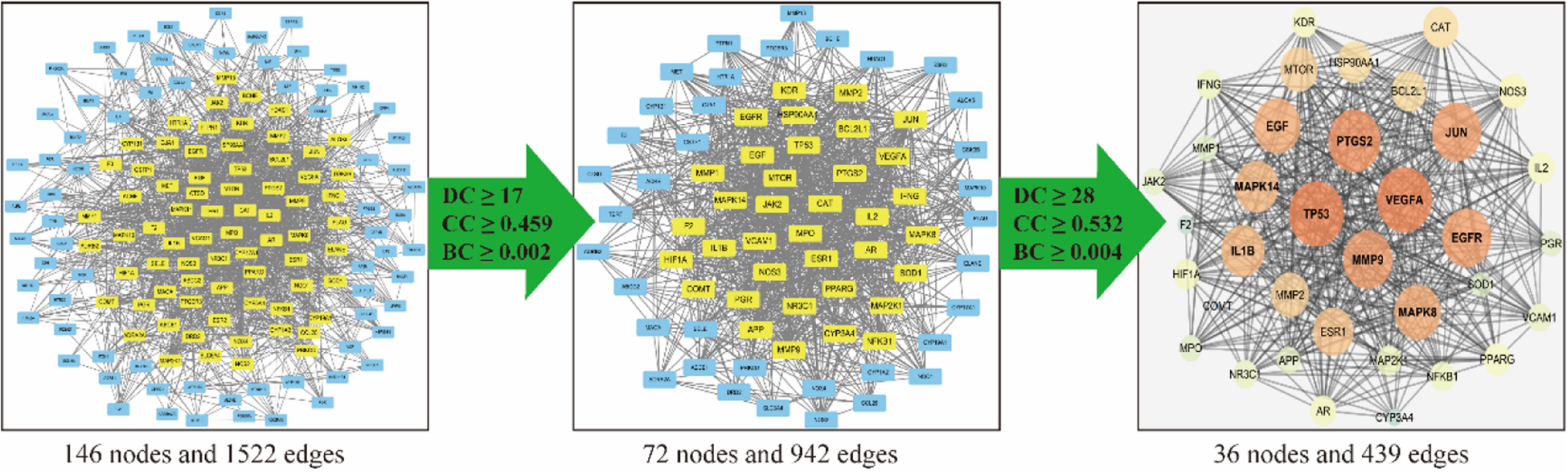
The whole screening process for the PPI network through a topological method. In the third image, the bigger size and brighter color represent higher DC value

**Fig. 5:**
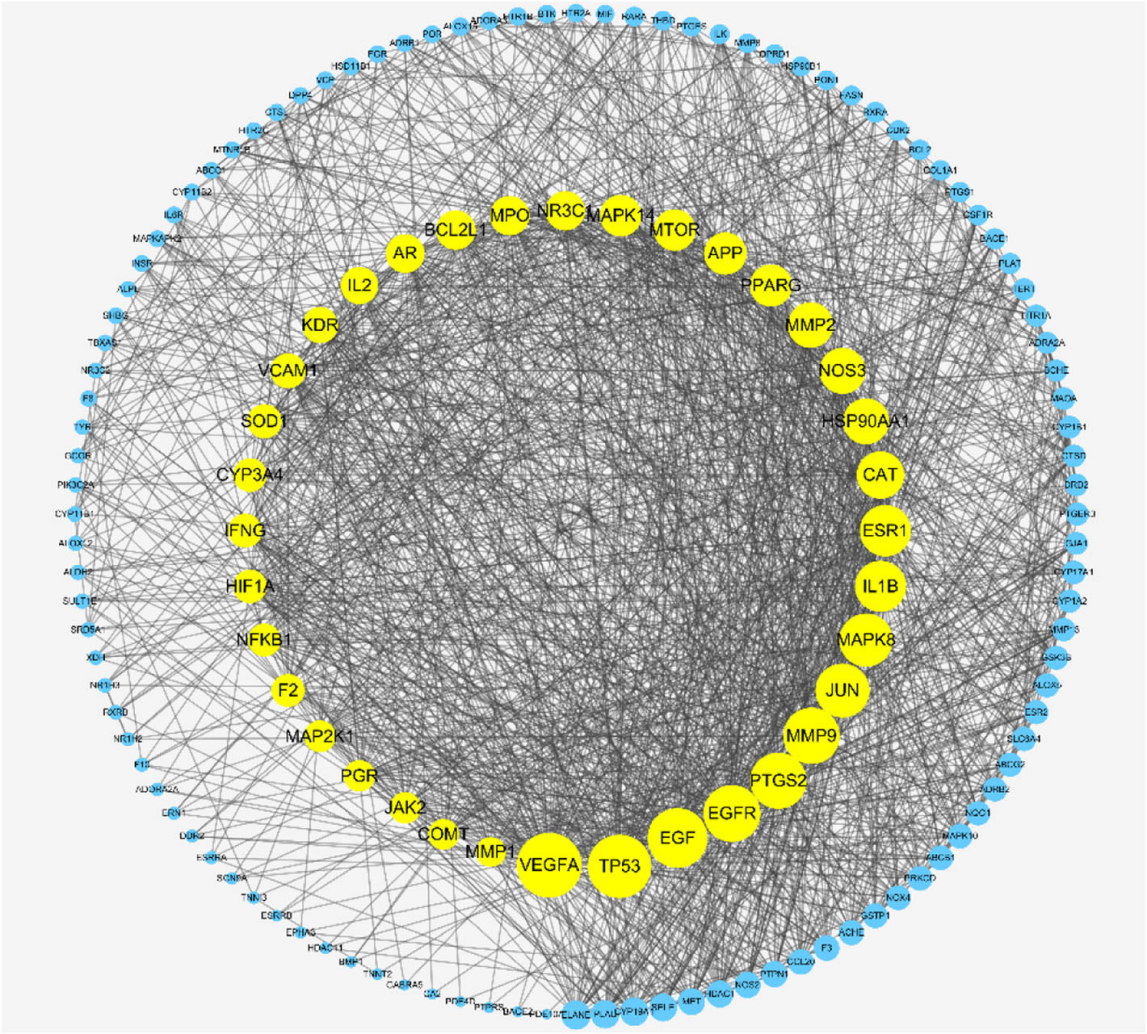
The whole PPI network for 146 nodes that yellow nodes represent 36 big hub genes. All the nodes ordered by size which depended on DC value

The data obtained from the String database were used to establish the PPI network for the 146 overlapping targets. In this network, there were 146 nodes and 1522 edges in total. Then, three main parameters, “degree”, “betweenness”, and “closeness”, were used as filters to select the key genes and build up the major hub nodes for the anti-OP effect of XLGB. The first screening threshold was degree≥17, closeness≥0.459 and betweenness≥0.002, which resulted in 72 nodes and 942 edges. Then, these 72 key nodes were further screened with the second threshold of degree≥28, closeness≥0.532 and betweenness≥0.004, and 36 nodes and 439 edges remained (Fig. 4). Among these nodes, VEGFA, TP53, EGF, EGFR, PTGS2, MMP9, JUN, MAPK8, IL1B, ESR1, CAT, HSP90AA1, NOS3, MMP2, PPARG, APP, MTOR, MAPK14, NR3C1, MPO, BCL2L1, AR, IL2, KDR, VCAM1, SOD1, CYP3A4, IFNG, HIF1A, NFKB1, F2, MAP 2 K1, PGR, JAK2, COMT and MMP1 were identified (Table 1). After sorting these 36 major hub nodes and 110 other nodes in descending order, we found that VEGFA (degree = 76), TP53 (degree = 74), EGF (degree = 69), EGFR (degree = 65), MMP9 (degree = 64), PTGS2 (degree = 64), JUN (degree = 61) and MAPK8 (degree = 60) were the key targets in this network (Fig. 5).

**Table 1:** Information of 36 hub targets

Uniprot ID	Gene Symbol	Description
P15692	VEGFA	vascular endothelial growth factor A
P04637	TP53	tumor protein p53
P01133	EGF	epidermal growth factor
P00533	EGFR	epidermal growth factor receptor
P35354	PTGS2	prostaglandin-endoperoxide synthase 2
P14780	MMP9	matrix metallopeptidase 9
P05412	JUN	Jun proto-oncogene, AP-1 transcription factor subunit
P45983	MAPK8	mitogen-activated protein kinase 8
P01584	IL1B	interleukin 1 beta
P03372	ESR1	estrogen receptor 1
P04040	CAT	catalase
P07900	HSP90AA1	heat shock protein 90 alpha family class A member 1
P29474	NOS3	nitric oxide synthase 3
P08253	MMP2	matrix metallopeptidase 2
P37231	PPARG	peroxisome proliferator activated receptor gamma
P05067	APP	amyloid beta precursor protein
P42345	MTOR	mechanistic target of rapamycin kinase
Q16539	MAPK14	mitogen-activated protein kinase 14
P04150	NR3C1	nuclear receptor subfamily 3 group C member 1
P05164	MPO	myeloperoxidase
Q07817	BCL2L1	BCL2 like 1
P10275	AR	androgen receptor
P60568	IL2	interleukin 2
P35968	KDR	kinase insert domain receptor
P19320	VCAM1	vascular cell adhesion molecule 1
P00441	SOD1	superoxide dismutase 1
P08684	CYP3A4	cytochrome P450 family 3 subfamily A member 4
P01579	IFNG	interferon gamma
Q16665	HIF1A	hypoxia inducible factor 1 subunit alpha
P19838	NFKB1	nuclear factor kappa B subunit 1
P00734	F2	coagulation factor II, thrombin
Q02750	MAP 2 K1	mitogen-activated protein kinase kinase 1
P06401	PGR	progesterone receptor
O60674	JAK2	Janus kinase 2
P21964	COMT	catechol-O-methyltransferase
P03956	MMP1	matrix metallopeptidase 1

**Fig. 6:**
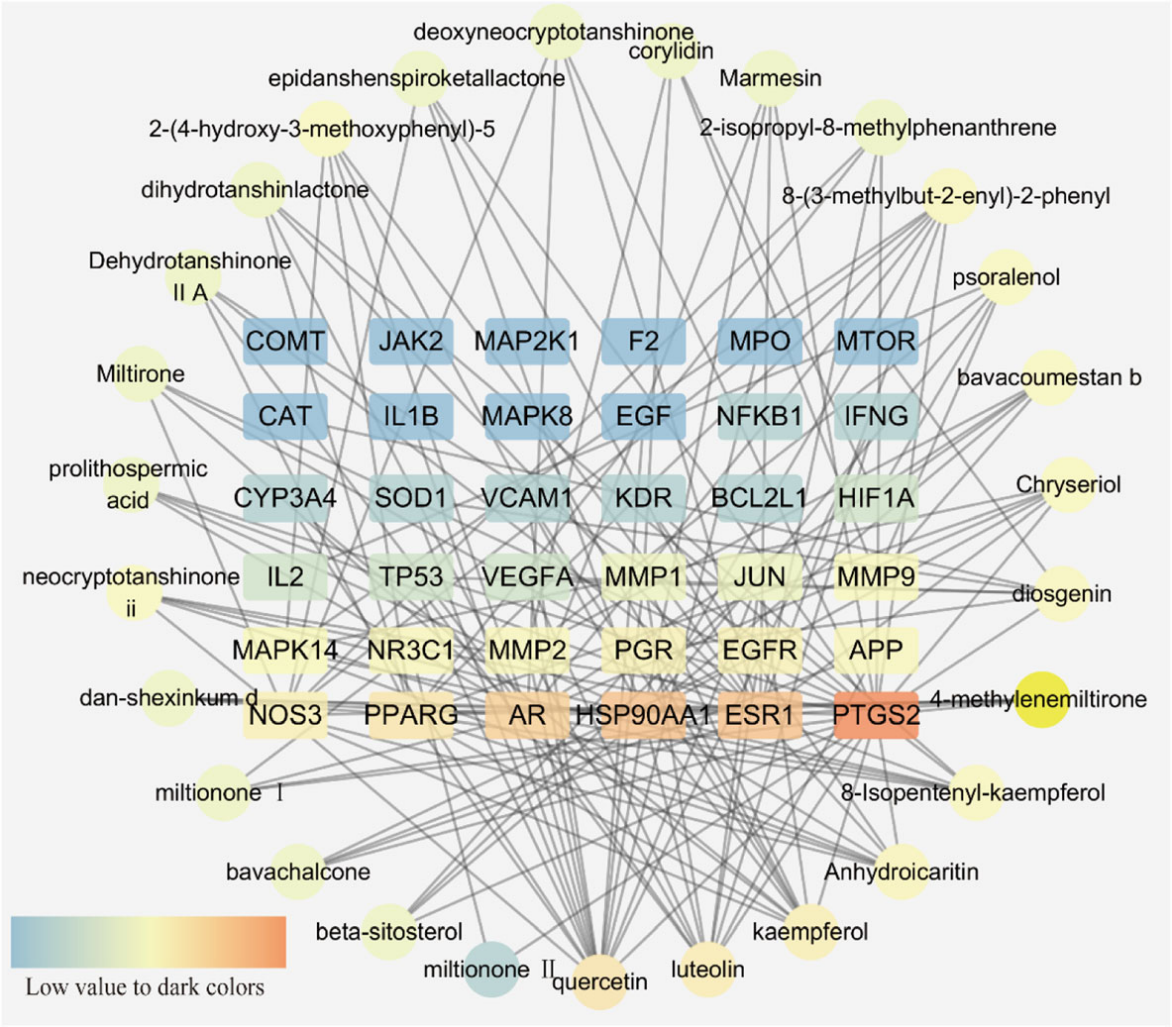
The network for big hub genes-comounds connection. The square nodes are the hub genes and round ones represent compounds. And all nodes’ color changes according to their degree value

Then, we further built up the major hub node-compound network (Fig. 6) based on these 36 key targets. This network included 27 compound nodes and 36 major hub target nodes. Subsequently, we reordered these compound nodes in descending order and found that quercetin was related to 17 major hub genes, luteolin to 12 genes, kaempferol to 11 genes, anhydroicaritin to 8 genes and diosgenin to 7 genes.

### GO biological process enrichment analysis

**Fig. 7:**
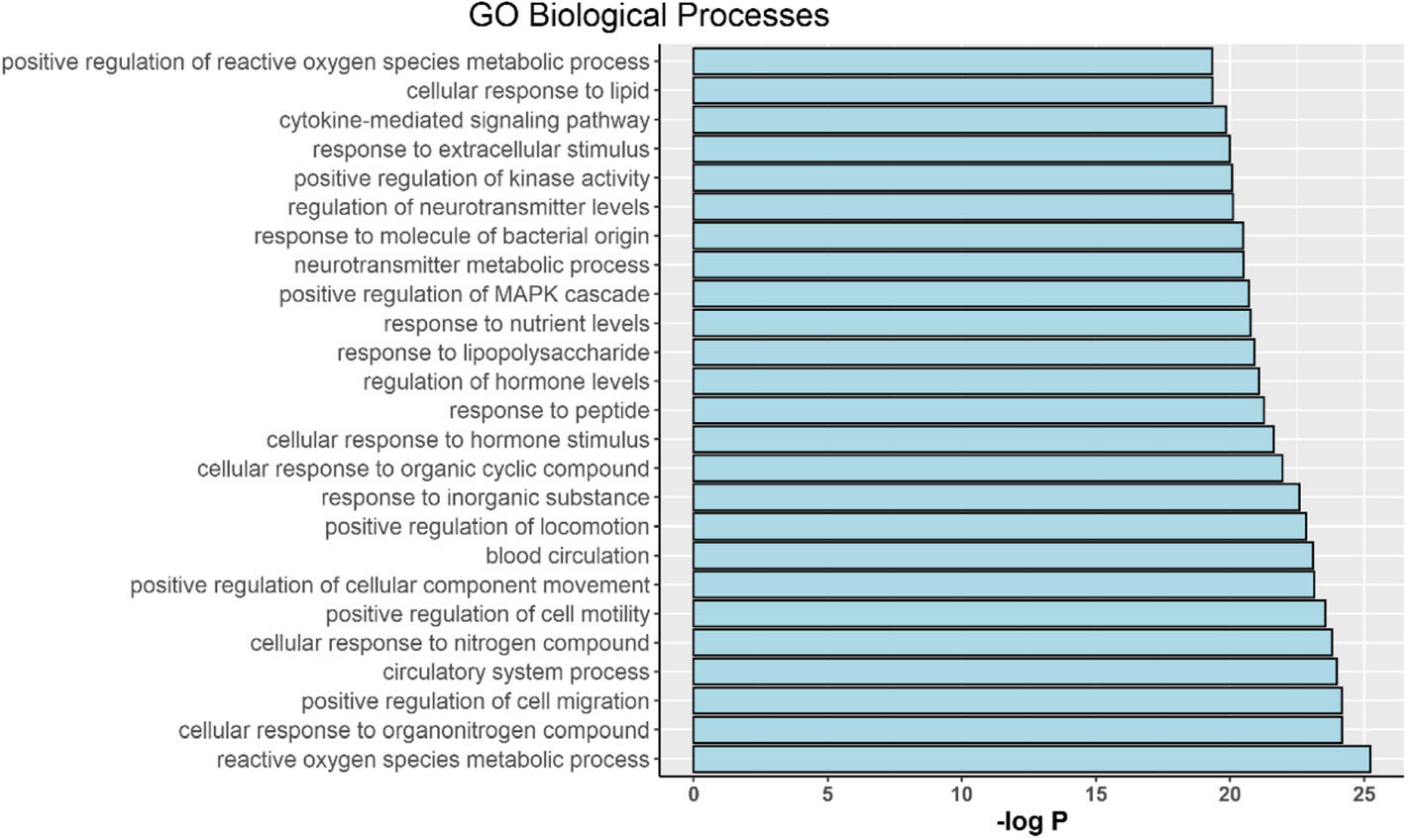
Biological processes enrichment for the top 25 processe

After sorting 343 Biological processes (BP) terms in ascending order of logP value, we found that the top 25 biological processes (Fig. 7) were mainly concentrated in the categories reactive oxygen species metabolic process, cellular response to organonitrogen compound and positive regulation of cell migration. Regarding the positive regulation of cell migration, the categories were positive regulation of cell migration (GO: 0030335), positive regulation of cell motility (GO: 2000147), positive regulation of cellular component movement (GO: 0051272), positive regulation of locomotion (GO: 0040017), positive regulation of MAPK cascade (GO: 0043410), and positive regulation of kinase activity (GO: 0033674). For the reactive oxygen species metabolic process, we mainly found reactive oxygen species metabolic process (GO:0072593), neurotransmitter metabolic process (GO:0042133), regulation of neurotransmitter levels (GO:0001505) and positive regulation of reactive oxygen species metabolic process (GO:2000379). Regarding the aspects of cellular response to organonitrogen compounds, we found cellular response to organonitrogen compounds (GO:0071417), cellular response to nitrogen compounds (GO:1901699) and response to peptides (GO:1901652). The remaining processes included cellular response to organic cyclic compound (GO:0071407), cellular response to hormone stimulus (GO:0032870), cellular response to lipid (GO:0071396), circulatory system process (GO:0003013), blood circulation (GO:0008015), cytokine-mediated signaling pathway (GO:0019221), regulation of hormone levels (GO:0010817), response to inorganic substance (GO:0010035), response to lipopolysaccharide (GO:0032496), response to molecule of bacterial origin (GO:0002237), response to nutrient levels (GO:0031667), and response to extracellular stimulus (GO:0009991). Based on these BP enrichment analyses, we found that the anti-OP effect of XLGB may result from its regulatory effect on reactive oxygen species, organonitrogen compounds and cell migration.

### KEGG pathway enrichment analysis

**Fig. 8:**
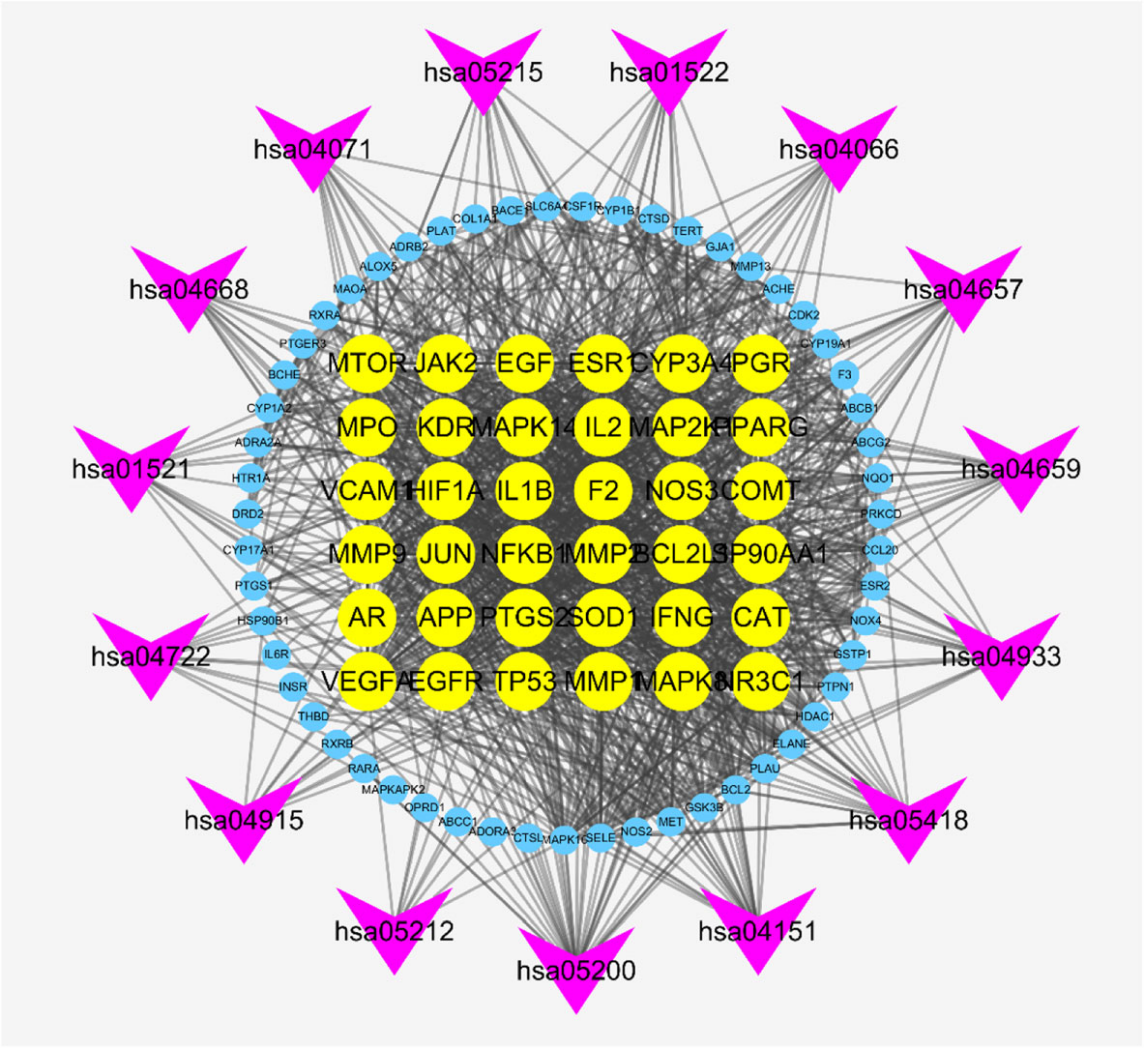
The targets-pathway network of XLFB for treating OP. The circle node represent genes and the yellow ones are the big hub genes. The arrow-like nodes represent the top 15 pathways

We further conducted KEGG pathway enrichment of 146 overlapping genes to determine the potential therapeutic mechanism of the XLGB capsules for OP. Then, we sorted 15 pathways based on the logP value; these pathways included pathways in cancer (hsa05200), fluid shear stress and atherosclerosis (hsa05418), AGE-RAGE signaling pathway in diabetic complications (hsa04933), Th17 cell differentiation (hsa04659), PI3K-Akt signaling pathway (hsa04151), IL-17 signaling pathway (hsa04657), prostate cancer (hsa05215), endocrine resistance (hsa01522), HIF-1 signaling pathway (hsa04066), EGFR tyrosine kinase inhibitor resistance (hsa01521), TNF signaling pathway (hsa04668), sphingolipid signaling pathway (hsa04071), estrogen signaling pathway (hsa04915), neurotrophin signaling pathway (hsa04722) and pancreatic cancer (hsa05212) (Table 2). We also constructed a target-pathway network based on the XLGB targets enriched in each pathway (Fig. 8).

**Table 2:** Information of 15 pathways

Term ID	Description	LogP	Count
hsa05200	Pathways in cancer	−26.20	32
hsa05418	Fluid shear stress and atherosclerosis	−25.80	23
hsa04933	AGE-RAGE signaling pathway in diabetic complications	−19.57	17
hsa04659	Th17 cell differentiation	−17.43	16
hsa04151	PI3K-Akt signaling pathway	−16.96	23
hsa04657	IL-17 signaling pathway	−16.88	15
hsa05215	Prostate cancer	−14.23	13
hsa01522	Endocrine resistance	−13.66	13
hsa04066	HIF-1 signaling pathway	−13.36	13
hsa01521	EGFR tyrosine kinase inhibitor resistance	−13.26	12
hsa04668	TNF signaling pathway	−11.59	12
hsa04071	Sphingolipid signaling pathway	−11.13	12
hsa04915	Estrogen signaling pathway	−10.72	11
hsa04722	Neurotrophin signaling pathway	−9.79	11
hsa05212	Pancreatic cancer	−9.76	9

## Discussion

XLGB capsules were shown to alleviate symptoms in osteoporosis patients and reverse bone loss in Ovarietomized (OVX) mice, which may result from the interaction between its active compounds and related osteoporosis targets. Thus, we investigated this potential mechanism through a network pharmacology method.

Based on the major hub gene-compound network (Fig. 6), we found that several key compounds, including quercetin, luteolin, kaempferol, anhydroicaritin, and diosgenin, play dominant roles in this network. Through a literature search, quercetin was shown to improve the bone mass and biomechanical properties of OVX rats, which is closely related to its protective effect against TNF-α-induced impairments in bone marrow mesenchymal stem cells [35]. Similarly, luteolin was shown to increase bone formation in glucocorticoid-induced osteoporosis by reducing the excess production of reactive oxygen species as well as promoting the proliferation and differentiation of osteoblasts [36]. Kaempferol has also been found to prevent osteoporosis-induced bone loss in vivo and in vitro, which results from its regulatory effect on the mTOR pathway [37]. Anhydroicaritin also has an inhibitory effect on RANKL-induced osteoclast differentiation, which leads to improved bone loss in osteoporosis caused by diabetes mellitus [38]. Similar to the above compounds, diosgenin can improve the bone mineral density of OVX rats through depressing the expression of RANKL and promoting OPG [39]. These active compounds may lay the foundation for the promising anti-osteoporosis effect of XLGB capsules.

On the basis of the analysis of the target-pathway network (Fig. 8) as well as BP enrichment (Fig. 7), we speculate that the potential mechanism of XLGB is probably related to its regulation of several biological processes, including reactive oxygen species, organonitrogen compounds and cell migration.

### Reactive oxygen species

**Fig. 9:**
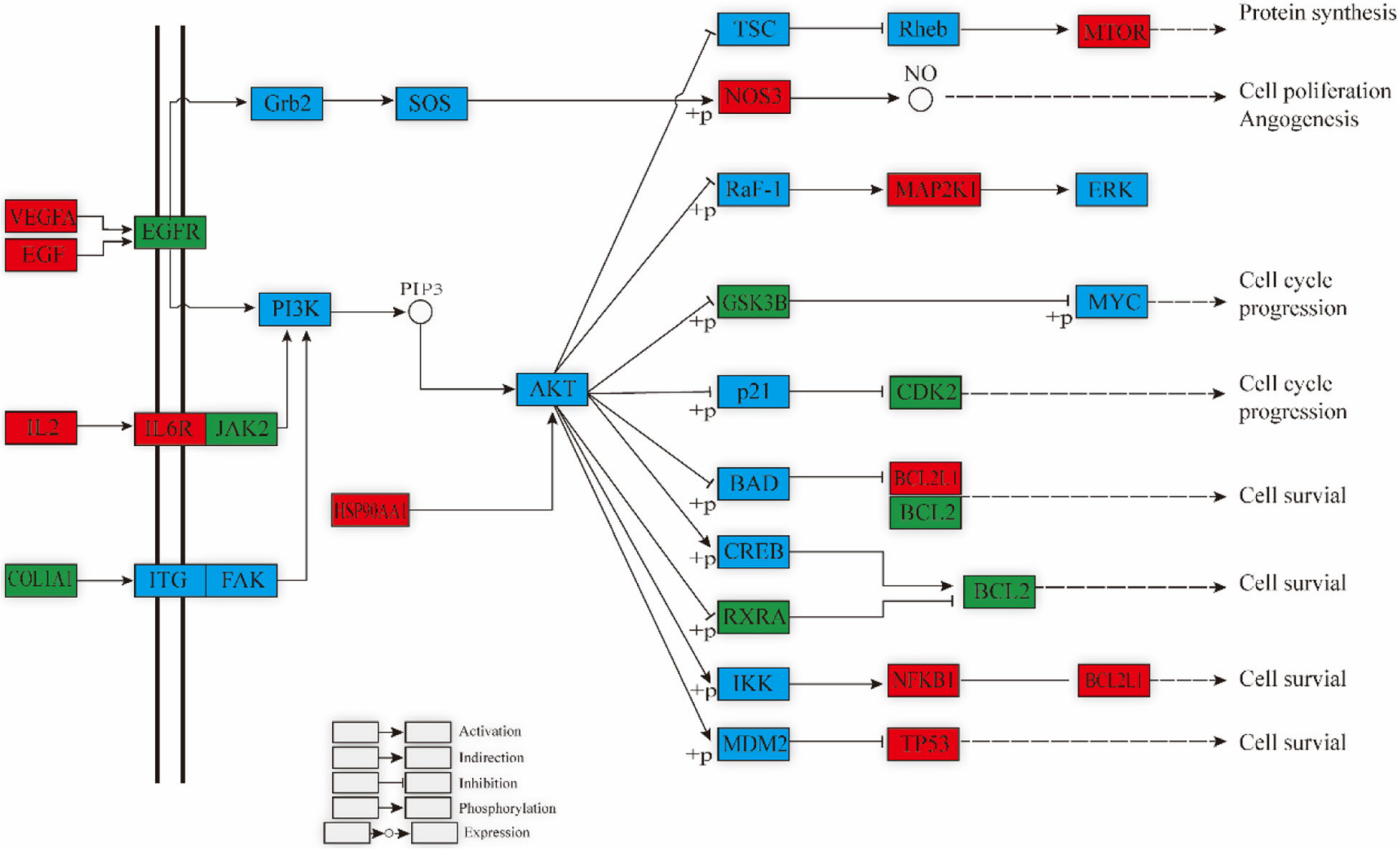
PI3K-Akt signaling pathway influenced by XLGB. The red nodes represent the hub genes, the green nodes represent other genes and the blue nodes is the genes of this pathway

Osteoporosis is a skeletal disease that primarily results from excessive osteoclast activity or deficient osteogenesis. Reactive oxygen species (ROS), produced during aerobic respiration, have been found to play a critical role in regulating cell proliferation, apoptosis, migration and differentiation [40], as well as in the regulation of osteoclasts and osteoblasts [41, 42]. EGFR and MTOR are overlapping genes between the reactive oxygen species and the PI3K-Akt signaling pathways based on the results of BP and KEGG pathway enrichment (Fig. 9). The PI3K-Akt-mTOR signaling pathway was first found in tumor cells and was shown to regulate numerous cell functions. MTOR, a downstream molecule of Akt, controls the metabolism of sugar and fat, as well as mRNA translocation to promote protein expression. This pathway has been proven to be related to the promotion of osteogenesis through upregulating the proliferation and differentiation of bone marrow mesenchymal stem cells [43–45]. Furthermore, Jin et al. demonstrated that the alleviation of the impaired PI3K/AKT signaling pathway induced by ROS was linked to its effect against osteoporosis through reducing H2O2-induced cell death in MC3T3-E1 cells. Epidermal growth factor (EGF) receptor (EGFR) serves as a key regulator molecule for osteoblast functions. Previous studies have shown that the activation of the EGFR-Akt-mTOR pathway can effectively protect osteoblasts against dexamethasone, which increases the ROS production in osteoblastic cells [46].

MAPK14 is a common gene in reactive oxygen species pathways, the PI3K-Akt signaling pathway and the TNF signaling pathway. MAPKs have important roles in cell proliferation, differentiation and apoptosis, as well as in regulating inflammation [47]. ROS can lead to serious periodontal tissue destruction by promoting osteoclastic bone resorption, which is closely related to the protein expression of MAPKs and NF-kB [48]. Srinivasa et al. found that the ROS-mediated facilitation of osteoclast differentiation and resorption can be reversed through inhibiting the NF-κB and calcineurin-NFAT pathways [49]. The activation of MAPKs also plays a key role in osteoclastogenesis induced by RANKL. However, this ROS-directed upregulation of MAPK and NF-κB signaling can be attenuated through strengthening nuclear factor-erythroid 2-related factor 2 [50].

Therefore, XLGB capsule’s regulatory effect on ROS is possibly due to the regulatory function of luteolin, diosgenin and anhydroicaritin. For example, luteolin, originally originating from *Epimedii herba* and *Radix Salviae*, may promote the expression of EGFR and then activate the EGFR-Akt-mTOR pathway to improve osteoblast functions. Similarly, diosgenin may also negatively regulate MTOR and subsequently activate the PI3K-Akt-mTOR signaling pathway to improve the osteoblastic differentiation of bone marrow mesenchymal stem cells. Moreover, anhydroicaritin may have an adverse effect on MAPK14, which further inhibits the PI3K-Akt signaling pathway and ultimately alleviates osteoporosis symptoms.

### Positive regulation of cell migration

Bone mass maintenance depends upon bone formation, which is mediated by mesenchyma stem cell (MSC)-derived osteoblasts. However, the imbalance of bone formation and resorption induced by osteoblasts and osteoclasts, respectively, will cause bone diseases such as osteoporosis. MSC migration is the early stage of bone formation, as MSCs differentiate into osteoblasts after they transmigrate to the bone surface [51]. Therefore, abnormal MSC migration will influence bone homeostasis. Previous studies have found that the migration of MSCs extracted from osteoporosis patients decreased dramatically, and it was also reduced significantly in aged and OVX rats [52, 53]. In general, insufficient migration of MSCs may be one of the vital mechanisms underlying osteoporosis, which is possibly a breakthrough for osteoporosis treatment.

EGF and EGFR were the overlapping targets between the cell migration biological process and PI3K-Akt signaling pathway. EGF and EGF-like ligands are effective chemoattractants for mesenchymal progenitors as well as for some epithelial cells, which are based on the interaction with the EGF receptor (EGFR) to promote the Akt pathway and ultimately increase MSC migration [54]. Furthermore, IL-1β and CCL20 are enriched in the IL-17 and TNF signaling pathways. Previous studies have demonstrated that some cytokines and chemokines, such as CCL20, can promote MSC migration. IL-1β, produced in different tissues, also promotes the migration and adhesion of MSCs by stimulating the secretion of CCL20 and other chemokines [55, 56]. As an important member of the matrix metalloproteinase (MMP) family, MMP9 is also enriched in the IL-17 and TNF signaling pathways. Each MMP family protease digests certain components of the extracellular matrix. In addition, MMP9 has a positive effect on promoting the migration of MSCs. Several studies have proven that upregulating MMP9 can increase MSC proliferation and migration [57, 58]. Notably, PI3K and AKT, as important migration-promoting factors for various cells, are related to the regulation of rBMSC migration, and the PI3K/Akt pathway contributed significantly to the increased secretion of MMP9.

In XLGB capsules, ingredients that act on these genes include luteolin, bavacoumestan B, psoralenol and bavachalcone. Based on these important components, XLGB may play a key role in regulating the migration of MSCs and then alleviate the symptoms of osteoporosis.

### Cellular response to organonitrogen compounds

This cellular response to organonitrogen compounds is related to the cell state or activity change after stimulation with a compound containing at least one carbon-nitrogen bond. Bisphosphonates (BPs) are the most frequently used treatments for osteoporosis to inhibit excessive osteoclast resorption activity. There are two different kinds of BPs that restrain osteoclast resorption. Non-nitrogen-containing BPs induce osteoclast apoptosis by inhibiting adenosine triphosphate-dependent enzymes. However, nitrogen-containing bisphosphonates (N-BPs) suppress the activation of farnesyl diphosphate synthase, which is necessary for osteoclast differentiation [59]. Notably, BPs containing nitrogen increased the inhibitory potency by 10–10,000 times compared to non-nitrogen-containing BPs [60]. In addition, N-BPs also inhibit preosteoclasts by inhibiting the PI3K/Akt pathway [61].

In the case of this organonitrogen compound response process, NF-kB1 is enriched in the PI3K-Akt, TNF, TH17, IL17, HIF and fluid shear signaling pathways. NF-κB is a critical transcription factor composed of Rel proteins, NF-κB1 and NF-κB2. NF-κB signaling activation is one of the downstream events after the stimulation of RANKL, which induces the differentiation and bone resorption of osteoclasts [62]. The XLGB formula may exert its anti-osteoporosis effect through regulating the cell response towards organonitrogen compounds and then inhibiting the expression level of NF-κB, eventually reducing the differentiation and bone resorption of osteoclasts.

## Conclusion

By utilizing network pharmacology, we explored the potential targets of XLGB compounds and investigated the underlying mechanism of their anti-osteoporosis effects, which may be based on three key biological processes: the inhibition of reactive oxygen species, promotion of the organonitrogen compound response and cell migration. According to the KEGG pathway enrichment results, we found that the PI3K-Akt signaling pathway is the main pathway when XLGB capsules are used to treat osteoporosis. Thus, we believe that the anti-osteoporotic effect of XLGB is mainly based on its direct or indirect regulation of the above potential targets and pathways, and our results provide promising directions for future research, which is essential to reveal the exact regulatory mechanisms.

## Data Availability

All major data used to support the findings of this study have been deposited in the figshare repository (10.6084/m9.figshare.11341358.v1). The datasets supporting the conclusions of this article are available in public databases from TCMSP, TCMID, Swiss Target Prediction, GeneCards, CTD, TTD, and PharmGKB.

## Abbreviations

BP: Biological processes
DL: Drug-likeness
GO: Gene ontology
KEGG: Kyoto encyclopedia of genes and genomes
OB: Oral bioavailability
OP: Osteoporosis
TCM: Traditional Chinese medicine
OVX: Ovarietomized
ROS: Reactive oxygen species
MSC: Mesenchyma stem cell
YYH: *Epimedium sagittatum* maxim
DS: *Salvia miltiorrhiza* bunge
DH: *Rehmannia glutinosa* steud
XD: *Dipsacus inermis* wall
ZM: *Anemarrhena asphodeloides* bunge
BGZ: *Psoralea corylifolia* L.
